# Supplementary material for: Financial Toxicity in Swiss Cancer Patients Treated with Proton Therapy: An Observational Cross-Sectional Study on Self-Reported Outcome
Source: Cancers (Basel). 2023 Nov 21;15(23):5498. doi: 10.3390/cancers15235498 (PMC10705792; doi:10.3390/cancers15235498)
Supplement: Supplementary file 1 [file cancers-15-05498-s001.zip › cancers-2657742-supplementary.pdf]

Tables

Table S1: Basic questionnaire

**Basic questionnaire (interview)**		
**Employment**		
	Not gainfully employed	
	Employed	
	Self -reliant	
	Unemployed	
	Recipients due to illness	
	Retired	
**Workload before illness**	0-100%	
**Current workload**	0-100%	
**Residing during proton therapy**		
	Distance from home	Km
	Living during proton therapy	At home
		With relatives/friends
		In apartment/hotel/guesthouse
		other
**Reimbursement of housing costs by health insurance**		
	Yes	
	No	
**Transport**		
	Daily transport to proton facility	
		Own car
		Rental car
		Public transport
		Taxi
		Ambulance
		Other

**Reimbursement of transport costs by health insurance**		
	Yes, completely	
	Yes, partially	
	No	
**Health insurance**		
	Cost coverage of proton therapy	
		Yes, completely
		Yes, partially
		No
	Franchise level	
	Amount of own charge for proton therapy	
	Limit of own charge	
		Yes, amount of the limit
		No
**Education level (for the caregivers: mother and father separately)**		
	Secondary school	
	Vocational training	
	High school	
	University/applied sciences	

Table S2: Cost diary

**Medical costs**		**CHF**
	Medication	
	Stationary stay in hospital	
	Ambulant medical consultation	
	Ambulant therapy	
	Nursing aids	
	Medical services	
	Other costs	
**Non-medical costs**		
	Round trips to proton therapy (PT) facility	
	Accommodation during PT	
	External meals due to PT	
	Phone calls due to PT	
	Household help due to PT	
	Leisure activities due to PT	
	Other costs	

Table S3: COmprehensive Score for financial Toxicity (COST) – Functional Assessment of Chronic Illness Therapy (FACIT). Items 2, 3, 4, 5, 8, 9, and 10 were reverse scored. The lower the score, the worse the financial toxicity.

		**Not at all**	**A little bit**	**Somewhat**	**Quite a bit**	**Very much**
**FT1**	I know that I have enough money in savings, retirement, or assets to cover the costs of my treatment	0	1	2	3	4
**FT2**	My out-of-pocket medical expenses are more than I thought they would be	0	1	2	3	4
**FT3**	I worry about the financial problems I will have in the future as a result of my illness or treatment	0	1	2	3	4
**FT4**	I feel I have no choice about the amount of money I spend on care	0	1	2	3	4
**FT5**	I am frustrated that I cannot work or contribute as much as I usually do	0	1	2	3	4
**FT6**	I am satisfied with my current financial situation	0	1	2	3	4
**FT7**	I am able to meet my monthly expenses	0	1	2	3	4
**FT8**	I feel financially stressed	0	1	2	3	4
**FT9**	I am concerned about keeping my job and income, including work at home	0	1	2	3	4
**FT10**	My cancer or treatment has reduced my satisfaction with my present financial situation	0	1	2	3	4
**FT11**	I feel in control of my financial situation	0	1	2	3	4
**FT12**	My illness has been a financial hardship to my family and me	0	1	2	3	4

Table S4: Questionnaire for Coping Strategies

I/we have to reduce our expenses for leisure activities (e.g. vacation, external meals)	Yes	No
I/we have to reduce our expenses for livelihood (e.g. food, clothing)		
I/we have to spend our savings or parts of them		
I/we have to borrow money		
I/we have to sell our property or parts of it		
My partner or I have to work more		
Other		

Table S5

Linear regression analysis of the COST score of the whole cohort (n=146), including the site of treatment, concomitant chemotherapy and treatment duration as explanatory variables.

		Estimate	Lower 95% CI	Upper 95% CI	p-Value
Intercept		30.29	21.31	39.27	< 0.0001
Site of treatment extracranial					
	Brain/Skull base	2.16	-1.97	6.25	0.30
	Head and Neck	1.66	-4.11	7.42	0.57
Concomitant Chemotherapy yes vs. no		-0.32	-4.65	4.01	0.88
Treatment duration (days)		-0.08	-0.29	0.12	0.42
